# Thinking about Later Life: Insights from the Capability Approach

**DOI:** 10.1007/s12126-018-9323-0

**Published:** 2018-02-15

**Authors:** Manik Gopinath

**Affiliations:** School of Health, Wellbeing and Social Care, Faculty of Wellbeing, Education and Language Studies, The Open University, Milton Keynes, UK

**Keywords:** Social constructions, Diversity, Heterogeneity, Relational ontology, Critical gerontology

## Abstract

A major criticism of mainstream gerontological frameworks is the inability of such frameworks to appreciate and incorporate issues of diversity and difference in engaging with experiences of aging. Given the prevailing socially structured nature of inequalities, such differences matter greatly in shaping experiences, as well as social constructions, of aging. I argue that Amartya Sen’s capability approach (2009) potentially offers gerontological scholars a broad conceptual framework that places at its core consideration of human beings (their values) and centrality of human diversity. As well as identifying these key features of the capability approach, I discuss and demonstrate their relevance to thinking about old age and aging. I maintain that in the context of complex and emerging identities in later life that shape and are shaped by shifting people-place and people-people relationships, Sen’s capability approach offers significant possibilities for gerontological research.

## Introduction

Critical gerontologists have long argued that social constructions of old age that speak to ‘ideals’ of aging are problematic (Estes et al. 2003; Katz and Calasanti 2015; Minkler and Fadem 2002; Rubinstein and de Medeiros 2015; Stephens 2016). For instance, they contend that such bodies of knowledge neither sufficiently acknowledge and account for diversity and difference in terms of people and contexts, nor give due consideration to the plural nature of things (including identities) that might matter more or less in growing old.

Critically engaging with social constructions of aging matters. It matters because how we think about ‘old age’ and ‘aging’ has a bearing on the design and practice of social policy. It matters, because it has implications for those who do not fit these narrow criteria; or for those who cannot play (if they so wished) the socially valued roles and lifestyles advocated by policy and discourse. It also matters because as ‘social constructions’ there is the potential to change them for the better (Phillipson and Walker 1987 as cited in Bernard and Scharf 2007). However, as scholars in Global North (Baars et al. 2013; Holstein and Minkler 2007; Wilson 2001) rightly note, this requires researchers to develop frameworks that can address issues of diversity and difference both in terms of people and contexts. Crucially, these scholars also recognize the need for frameworks that can attend to older peoples’ own experiences and meanings of aging within the context of any social construction. In this commentary, I argue that Amartya Sen’s capability approach (2009) has the potential to offer a conceptual framework that incorporates human diversity, heterogeneity amongst people, and plural values; as well as simultaneously locating the individual in the social.

The remainder of the commentary is laid out as follows. In the next section, I set out the key concepts of the capability approach. This is followed by a detailed discussion around how core features of the approach offer a conceptual resource to critically and usefully think about old age and later life. Applying the capability approach is not without its challenges and these will be discussed next. I conclude by reflecting on the potential that the capability approach offers for thinking about old age and aging.

## Capability Approach: Key Concepts

Sen’s capability approach “is a broad normative framework for the evaluation and assessment of individual well-being and social arrangements, the design of policies, and proposals about social change in society” (Robeyns 2005a, p.94). ‘Capabilities’ and ‘functionings’ are two key concepts of the capability approach. Capabilities refer to the ‘genuine opportunities’ that people have to achieve various valued functionings (Sen 1993). In making evaluations, the quality, quantity and diversity of available opportunities also matter (Crocker David 1998). Functionings are various things a person may do or be. These could include *ways of being*, such as sleeping, being literate, being content, and being a researcher; as well as *acts of doing*, such as going to the theatre, caring, writing a book, cooking, and gazing out of the window. So, for example, living in a warm and damp-proof house (in a cold climate) is a functioning; and the genuine opportunity that a person has to live in a warm and damp-proof house is the capability for that functioning (Robeyns 2016).

Human beings and their capabilities are end concerns of the capability approach (Alkire 2002). Sen (2009) argues for a shift in evaluations of wellbeing, quality of life, disadvantage, and inequality, from the space of ‘resources’ and ‘preferences’ to the space of ‘capabilities’ (Sen 2009). The relational ontology (Smith and Seward 2009) of the capability approach incorporates a relational conception of people and their capabilities situated within the social. People are, therefore, seen as socially situated beings and their capabilities understood as socially shaped. As such, the capability approach demands a broad informational focus to include both the person and context (Clark 2008). Two core features of the capability approach that can contribute to thinking about old age and aging are: attention to human diversity, and concern with values.

## Centrality of Human Diversity

Human diversity is central to the capability approach. Sen writes, “*human diversity is no secondary complication to be ignored, or to be introduced later on; it is a fundamental aspect of our interest in equality*” (Sen 1992, xi). The novelty of the approach lies not in acknowledging the existence of human diversity, but in incorporating this aspect in developing an account of wellbeing and/or design of social policies. Expanding on diversity, Sen (2009) lists at least five sources of diversity ranging from individual to contextual factors. These extend from heterogeneity amongst people, for instance, in terms of attributes and personal circumstances including age, gender, disability or illness, material resources amongst other things, to variations in the context, such as policies, the physical environment that one inhabits, social relationships, socio-cultural norms, discourses and beliefs which may potentially affect, influence and/or shape a person’s capability. Sen (1992) emphasizes that such differences matter given the prevailing socially structured nature of inequalities that influence what people are able to do and to be. Conceptually and analytically, the capability approach permits theorizing old age in ways that give due consideration to group differences of gender, race, class, age, sexuality, of circumstances and contexts.

A major criticism of mainstream gerontological frameworks is their inability to appreciate and incorporate diversity when examining experiences of aging (Holstein and Minkler 2007). For example, experiences and voices of some groups of older people, such as women, ethnic and racial groups, the poor, and/or those with impairments, get subsumed within prevailing and dominant discourses of later life. Frequently, they become excluded because of essentialising the categories of, and normative assumptions about, old age, cultural norms of independence and autonomy; and/or because of their material locations (Grenier 2005; Katz and Calasanti 2015; Wray 2003). For instance, research shows that, when internalized, cultural norms of independence can compound disadvantage for those who might experience multiple forms of inequality (Breheny and Stephens 2012). This can take various forms, such as declining the need for support and help from children where available to uphold the ideal of not being a burden (Portacolone 2013); and/or, continuing to live in a long term domestic home despite its undesirable implications for wellbeing.

Foregrounding of diversity within the capability approach suggests that it might be possible to frame or theorize ‘old age’ in non-essentialising ways. This is because the capability approach does not support normative conceptions, for instance, of what it means to age successfully or what should be a reference person. Its normative concern is with peoples’ capabilities. From a capability perspective, questions might be asked, for example, about the following:

- what are the valued capabilities identified by different groups of older people?
- under what circumstances and for whom social contexts enable and/or constrain capabilities including their identities?
- how do inequalities in health influence capabilities in later life and for whom?
- how does age and gender matter in shaping capabilities?
- how does social policy shape and support capabilities that older people have and value?
- what capabilities should be accounted for in social policy considerations?
- what assumptions about older people shape specific social policies and to what extent these serve to enable or constrain capabilities?

Taking account of diversity in the capability approach is not limited to incorporating differences solely in terms of age, gender, sexuality etc. The concept of ‘conversion factors’ offers another evaluative space to incorporate heterogeneities of person and context (Sen 2009). Conversion factors can be aspects of person (e.g. impairment, skills, emotions) or context (e.g. physical environment, climate, institutions, social and cultural norms, discourses, policies) that influence how particular resources and goods can (or cannot) be converted into capabilities (Robeyns 2005b). When thinking about capabilities for specific functionings, conversion factors do two things: firstly, they alert us to the possibility of gradations and variations in personal and/or contextual circumstances of different people; secondly, they promote a nuanced understanding about the capabilities that different people may have, in some instances, even for the same functioning (Sen 1992, 2009).

For example, extending working lives and raising pension ages may not support capability for work for those in poor health or with caregiving duties. For some, discriminatory employment practices and ageist attitudes may constrain their capability for employment despite eligibility as per chronological age. A mobility scooter may not be of much use for an older person to get around for a number of reasons: if the pavements are not wide enough (Gilroy 2006); if the person does not have the requisite skills; and/or gender perceptions or norms are skewed against women riding a mobility scooter. A capability lens can permit serious consideration of a range of social and personal constraints or enablers that shape experiences of aging without making assumptions about capabilities people have or nature of constraints they face.

The notion of personal conversion factors within the capability approach can also permit consideration of differences in bodies, such as a range of impairments or chronic illness (Mitra 2006). The capability approach’s relational ontology means that it can attend to the (aging) body within the context of the social (Terzi 2005). As several scholars note within gerontological research, discussions about the body have either conflated old age with disease and decline (Pickard 2014), or seen it through the lens of youth (Calasanti and Slevin 2001). Several scholars (Stephens 2016; Timonen 2016; Wilson 2001) have also expressed concerns about how certain conceptions of later life deny the challenges of aging with health and age-related impairments. Moreover, unequally structuring opportunities, which intersect impairments with other social locations, can negatively impact upon experiences of aging for some (Minkler and Fadem 2002; Oldman 2003).

Not taking into consideration the aging body also means relegating it to the biomedical realm (Twigg 2006), allowing the aging body and its impairment to be considered purely in functional terms, rather than exploring how function maps onto experience (Gilleard and Higgs 2014). The idea of personal conversion factors can encourage looking beyond biomedical conceptions to take account of, for example, diversity in bodily capacities. In accounting for interpersonal variations, arising from multiple combinations of personal and contextual factors, the capability approach can account for both heterogeneity and diverse social locations.

## Concern with Values but Not Imposition

The capability approach opens up space for valuing and giving voice to people’s own conceptions of what matters rather than making normative or a priori assumptions or as Calasanti (1996) notes labelling some as ‘special or deviant cases’. This derives partly from its ethically individualistic stance (Robeyns 2005b), in that it is concerned with people’s individual capabilities to choose and lead their lives in ways that matter to them, situated within their own social and cultural contexts. The emphasis in this approach on each person having and enjoying equal (moral) worth aligns with concerns of both feminist scholars (Robeyns 2008) and, more recently, scholars of gerontology (Stephens 2016). This because it permits a conceptual shift from normative ideals (e.g. successful aging, productive aging) and normative reference/evaluative standpoints (e.g., lens of male, healthy and able-bodied, specific racial perspectives, third/fourth age binary) to include voices of people who are often excluded.

The capability approach takes account of the need to value people’s own conceptions of what matters in several other ways. Sen (2009) acknowledges that some capabilities might be more generally valued but refuses to specify or endorse the content of valued capabilities, and as discussed later this raises challenges for empirical applications. Rather, he argues against imposing values by maintaining that “it is the people directly involved who must have the opportunity to participate in deciding what should be chosen, not local elites (political or religious) or cultural experts (domestic or foreign)” (Sen 1999, p.31–2).

Another way that the capability approach avoids imposition is by stressing capabilities over functionings while acknowledging that, in instances of acute deprivation, for example, it might be pragmatic to focus on functionings (Sen 2009). A capability lens on later life would primarily be concerned with whether people have genuine opportunities (taking note of a range of constraints, such as, ill health, mobility difficulties, availability of resources, social support, gendered nature of some social spaces, environmental barriers, discrimination and quality and quantity of available options) to participate in social and community life. It would not impose or prescribe one way of participating over another, leaving people free to realize (or not) the specific functioning of participating. Policy prescriptions of what should be valued and prioritized in later life are evident in the emphasis on civic engagement (Deeming 2009; Martinson and Minkler 2006). Research, however shows that such prescriptions do not respect other ways of living and being (including identities), that might be meaningful depending upon context (Clarke and Warren 2007; Raymond et al. 2014).

However, a concern with the person in the capability approach does not equate to the conception of a person as an active agent, a self-governing individual, responsible for self, which is often characterized by a narrow focus on autonomous choices and stable preferences (Entwistle and Watt 2013; Sointu 2005). Rather, a relational conception permits consideration of a wide range of constraints and enablers including, for example, personal skills and attributes, availability of social support, and institutional factors that shape opportunities, choices and choice making. Conceptually, in focusing on people’s capabilities, the approach permits rejection of binary conceptions of older people (e.g. as patients, consumers), and, ‘binary interpretations of agency as present or absent’ (Grenier and Phillipson 2013). In so doing, it steers clear of assigning older people a specific ‘social’ identity that smothers out possible other simultaneously held and valued identities (Smith and Seward 2009). From a capability perspective, social identities of those older people who choose not to or cannot aspire to normative ideals are, therefore, not fixed or framed. As such, the capability approach aligns with concerns amongst critical gerontologists (Biggs 2001; Wilson 2001) of the need to recognize identities as multiple, partial, and fluid.

Finally, the capability approach supports recognition of plural notions about what matters (Sen 2009). It further acknowledges that people may differ in the relative importance they attach to specific capabilities although, some capabilities (e.g., to be adequately nourished; to enjoy social relationships; to be respected) might be more generally widely valued. That there can be plural and diverse responses to value stems from recognition of the heterogeneity amongst people, including valued identities, and serves as a critique to singular notions of value as reflected in income or happiness. Recognition of plurality of values can also permit consideration of other dimensions, such as, spirituality and its significance for later life (Sadler and Biggs 2006). However, this does imply that evaluative considerations disregard the interconnected nature of capabilities, in that some capabilities (or lack of them) can be instrumental in enlarging and/or constraining other capabilities (Wolff 2009).

## Implications and Challenges

Though widely used in fields of study such as education, health, development, disability, gender and welfare economics, the capability approach is not without its criticisms (Clark 2008). At one end are critiques particularly within political philosophy. Framed within justice concerns, these criticisms question the viability and superiority of space of ‘capabilities’ over ‘resources’ (Pogge 2002). At the other end, within welfare economics are concerns with operationalization, measurement and aggregation of capabilities (Martinetti 2006). Others have raised concerns that the capability approach does not sufficiently take into consideration unjust global structures (Dean 2009), or that it limits itself to the individuals as moral unit of concern (Deneulin 2008), or that it does not tackle the question of negative capabilities (Qizilbash 1996).

A major concern shared by many scholars relates to the deliberate under-specification of what ‘capabilities’ should focus upon. The strength of the capability approach, i.e., not articulating a specific conception or content of a good life (Sen 2009), presents a methodological challenge for empirical research. It raises questions about identifying and selecting relevant capabilities (Alkire 2002). Whether selection of capabilities is to be done democratically or philosophically constitutes another area of disagreement and is a matter of ongoing debate and development (Byskov 2017). These concerns are not easy to address. Yet they are not insurmountable, as wider empirical applications of the capability approach suggest. Such applications use different methods for eliciting relevant capabilities that matter to groups and/or individuals. A common feature that these applications share is the emphasis on some form of democratic procedure and deliberation. Selection of capabilities depending upon the scale and purposes of the research can be achieved through various procedures: combining theoretical conceptions of a good life with participatory methods (Alkire 2002); democratic deliberation and debate (Vizard and Burchardt 2007); and/or through the basic criterion of ensuring that selected capabilities are ‘explicit, discussed and defended’, open to revision and development (Robeyns 2003).

In *Idea of Justice* (Sen 2009) Sen makes clear that the capability approach is not a ‘theory’ of justice. Being a framework and not a theoretical model with ‘various bits to be filled in’ (Sen 1993:48) permits users to complement the capability approach with other frameworks and methodologies according to purpose and context (Deneulin 2014). This in turn raises epistemological challenges, as analysis from a capability perspective will vary depending upon the choice of complementary frameworks and methodologies. For example, much depends on whether we complement such a framework with relational or personal notions of autonomy, notions of interdependence or independence, and/or how we conceptualize notions of responsibility and difference (Robeyns 2008). Feminist scholars acknowledge that capability approach gives due consideration to the person and their context. Yet from a feminist perspective it remains vulnerable to ‘androcentric biases’ (Robeyns 2008) unless the social is theorized to account for gendered nature of social relations that differentially impact capabilities of men and women. Calasanti (2010) with respect to later life presents a similar argument for theorizing gender to understand how gendered social relations differentially shape experiences of both men and women in later life.

In a similar vein, and rightly so, McMullin (2000) makes a case for incorporating age relations as a source of diversity. Age, can be one amongst many social locations that shape diverse experiences in later life. Age relations can socially structure meanings of old age, including bodies, as well as where intersections with other differences of, for instance, gender, bodily capacities and class are likely to shape diverse experiences (positive and negative) (Pain et al. 2000). The study by Moran et al. (2013) shows how assumptions about the use of services by older people in England (United Kingdom) shaped the size of their cash budgets, in contrast to younger groups. This in turn impacted how they could or could not use the budgets.

A ‘critical use’ of the capability approach that theorizes structural relations (e.g., of gender, age as applicable) would offer a more robust contextualization of the social and permit nuanced understanding of ‘……combinations of individual capacities and social context [that] result in particular capabilities’ (Smith and Seward 2009, p.228). The relational ontology of the capability approach indicates the possibility of complementing it with other relational perspectives, which recognize the socially embedded and situated nature of human existence. For example, thinking in terms of capabilities, and using relational perspectives on ‘place’ (Massey 1994; Wiles 2005) to conceptualize person-environment interactions, can illuminate a broad range of ways in which place(s) can enable and constrain capabilities.

Arguing for people’s own conceptions of what matters, the capability approach can lend itself to narrative and participatory methodologies for undertaking research with older people. If such methodologies are used critically, they can serve to undo ‘traditional sources of knowing and authority’ (Holstein and Minkler 2007, p.26). The information basis of the capability approach requires explicating the dynamic between situated nature of capabilities and people’s values thereby emphasizing a qualitative stance (Zimmerman 2006).

## Capability Approach and Aging Research

Unlike other fields, there are few applications of the capability approach within aging research. Of these, some focus on developing and validating a conceptual measure of wellbeing (Grewal et al. 2006) and living standards (Breheny et al. 2016). Such research shows that what older people value is the capability to achieve valued functionings: the inability to pursue capabilities negatively influences wellbeing. Other studies, drawing on insights from capability approach empirically highlight the narrow focus of current mainstream gerontological frameworks (Stephens et al. 2015). These suggest the need to pay attention to the material and social contexts of older people in understanding and supporting the health of older adults regardless of health status. Gilroy (2006) and Stephens (2016) separately drawing on the capability approach offer conceptual critiques of assumptions behind and implications of current aging discourses for wellbeing of older people. Zaidi (2008, 2011) investigates links between income and capabilities amongst older people in the European context. His work suggests that adequate assessments of wellbeing need to complement income measures with a range of other measures.

The capability approach offers a broad framework at the core of which is an ethical commitment to peoples’ concerns and quality of lives. Depending upon the context, purpose and interpretation, a capability framework can be used in a descriptive way to analyse how social arrangements influence capabilities or in a prescriptive way to drive forth social change and transformation (Alkire 2008; Deneulin 2014). In offering a sympathetic account, I am not seeking to establish the hegemony of capability approach. Instead, I seek to broadly and ambitiously sketch out, possibilities the capability approach as a conceptual resource, can offer gerontological research.

## Conclusion

In this commentary, I pick up on a longstanding critique of mainstream gerontological frameworks, focusing on how social constructions of old age that speak to ideals of aging are simultaneously homogenizing, exclusionary and de-contextualised. Setting out key concepts, I demonstrate how a capability lens offers gerontological scholars a general framework within which to critically engage with experiences of aging.

The capability approach takes seriously and can offer a complex, nuanced understanding of old age and aging that is attentive to diversity and contexts, as well as differing perspectives on values. Its concern with social arrangements demands a broad informational focus, requiring contextualised consideration of a range of social constraints on people’s capabilities to lead lives in ways that matter to them. A relational ontology permits a focus on processes and interactions thereby encouraging consideration of fluid and dynamic nature of interactions between the social and individual.

To conclude, a key question shaping research pursued by critical gerontologists is ‘*how best to bring together the ‘social’ and ‘individual’ in attending to experiences of aging?’* A capability lens with ‘various bits filled in’ is well positioned to address many of those challenges. How the capability framework and its features are employed might vary with the purpose and context of the research. To begin with, and at a minimum, it permits a language that is attentive to each (older) person without labelling and categorizing, which is a notable and consequential consideration in constructions of old age.
